# Heart Repair Gets New Muscle

**DOI:** 10.1371/journal.pbio.0030125

**Published:** 2005-03-15

When you think of the cell as the fundamental unit of life, it's not surprising that some organs deal with injury better than others. A flesh wound or muscle tear might hurt, but, assuming you are otherwise healthy, both will heal. The prognosis for a heart attack, on the other hand, is not so clear-cut. What accounts for the difference?

Skin cells reproduce regularly to replace dead cells, and simply increase production in the event of injury. Skeletal muscles recruit new muscle cells from a type of precursor cell within the muscle, called satellite cells, to repair a tear. Cardiac cells (cardiomyocytes), it has long been thought, appear to lack this capacity for self-renewal and repair, impeding the chances of a full recovery. That's why therapies derived from stem cells—which retain a unique ability to morph into any of the body's 200-plus cell types—hold such promise. But stem cells are a hot-button issue in the United States, complicating efforts to explore this promise.

Recent evidence suggests that the heart might harbor stem cells after all and that such cells can be transformed into cardiomyocytes. In a new study, Neal Epstein and colleagues report that cells isolated from the skeletal muscle of adult mice can turn into beating cardiomyocytes in a test tube within days of isolation—and without the addition of gene-altering drugs or special cardiac factors. When freshly isolated cells (called skeletal precursors of cardiomyocytes, or Spoc cells) are injected into the tail veins of mice with heart damage, they migrate to the damaged tissue and differentiate into cardiac muscle cells.

What distinguishes a cardiomyocyte from a skeletal muscle cell? Specialized cells produce unique proteins, allowing scientists to use those proteins as identifying markers. The so-called Spoc cells do not express any of the usual markers associated with either skeletal muscle satellite cells or partially differentiated skeletal muscle cells. By day 7 in culture, Spoc cells have undergone several rounds of cell division and have begun to express a (mostly) cardiac-specific protein, and have formed clusters of cardiac precursor cells, some of which beat. These precursors in turn express other cardiac-specific proteins.

Epstein and colleagues further divided Spoc-derived precursor cells into two groups based on whether or not they expressed another protein marker (Sca-1, a common marker found on blood stem cells). About 80% of cells without this protein differentiated into immature beating cells after proliferating for seven to ten days. They remained in an immature state (round and loosely attached) for over two months in culture, but differentiated into mature beating heart cells (elongated and adherent) when mixed with Sca-1 cells. The authors use video microscopy to track the cells' progression to beating cells, complete with contraction-generating thick myosin filaments that are “nearly identical” to those seen in developing cardiomyocytes. Epstein and colleagues also demonstrate that the Spoc cells are distinct from stem cells cultured out of bone marrow, heart, or fat tissue—sources of beating cells in other studies. The authors also injected these Spoc cells into mice with acute heart lesions to test the cells' ability to integrate into the damaged tissue. Many cells successfully migrated to and engrafted into the site of injury; some of these cells developed into cardiomyocytes. The cells showed a similar, though less robust, response to an older heart injury.[Fig pbio-0030125-g001]

**Figure pbio-0030125-g001:**
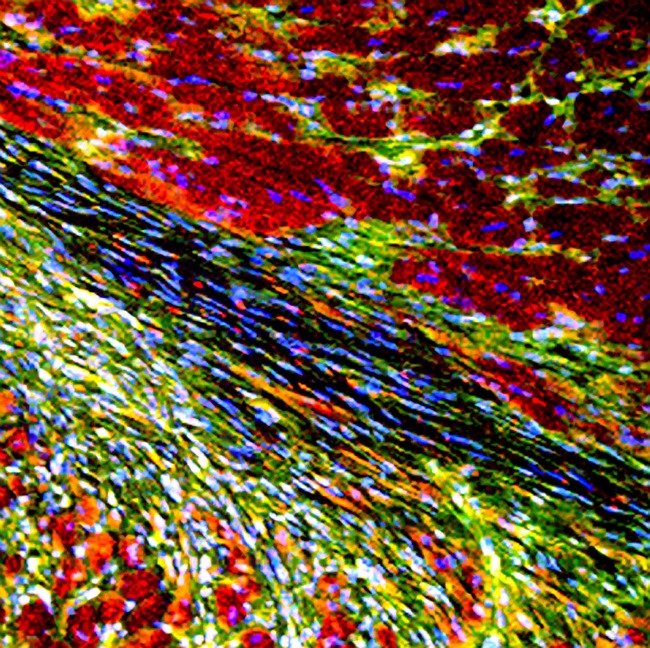
Spoc cells can help repair a damaged heart

Epstein and colleagues argue that Spoc cells are more likely to be precursors to cardiomyocytes than to be some other type of skeletal muscle stem cell. This is based on an absence of protein markers for skeletal muscle or skeletal satellite cells in Spoc cells, as well as the fact that Spoc-derived cells display spontaneous rhythmic beating and express cardiac markers, whether they are grown in a test tube or have migrated to injured hearts in study mice. The authors can't say why skeletal muscle would harbor cardiac stem cells or why so few of these cells pitch in to repair a cardiac injury. But for now, the Spoc cells provide a valuable tool for studying heart cell differentiation. And with time, they might prove an important resource for developing cell-based therapies for heart disease. See also the Primer “Alchemy and the New Age of Cardiac Muscle Cell Biology” (DOI: 10.1371/journal.pbio.0030131).

